# Machinability of the Thermoplastic Polymers: PEEK, PI, and PMMA

**DOI:** 10.3390/polym13010069

**Published:** 2020-12-26

**Authors:** Ying Yan, Yu Mao, Bo Li, Ping Zhou

**Affiliations:** Key Laboratory for Precision and Non-Traditional Machining Technology of Ministry of Education, Dalian University of Technology, Dalian 116024, China; 172375884@mail.dlut.edu.cn (Y.M.); libo11@mail.dlut.edu.cn (B.L.); pzhou@dlut.edu.cn (P.Z.)

**Keywords:** polymer, micromilling, surface quality, temperature

## Abstract

The thermoplastic polymer such as poly(methyl methacrylate) (PMMA), polyetheretherketone (PEEK), and polyimide (PI) is a kind of polymer material with properties of good mechanical strength. It has been widely used in the fields of aerospace, optical engineering, and microfluidics, etc. Thermoplastic polymers are considered to be one of the most promising engineering plastics in the future. Therefore, it is necessary to further study its mechanical properties and machinability, especially in ultra-precision machining. Furthermore, mechanical property and machinability were studied in this work. Through the dynamic mechanical analysis experiment, the elastic modulus and temperature effect of PMMA, PEEK, and PI are analyzed. In addition, the high-speed micromilling experiment is conducted to show that the surface roughness, burrs, and cutting chip characteristics in the high-speed micromilling process. In general, the surface quality of the brittle removal is generally better than that of the viscoelasticity state. The results show that PMMA, PEEK, and PI have good mechanical properties and machinability. Base on the results, the material will be in a viscoelastic state as the temperature increases. The surface quality of the brittle removal is generally better than the viscoelastic state.

## 1. Introduction

Polymers are widely used in industries such as the aerospace industry, optical engineering, and biological engineering industry due to the excellent properties such as low-density, corrosion resistance, low coefficient of friction, and the possibility of mass production [1,2,3,4,5,6]. Among the various types of polymers, thermoplastics are difficult to cut due to their characteristic properties such as low modulus of elasticity, low thermal conductivity, high coefficient of thermal expansion, and internal stress [7,8,9]. For the special application of polymer, the integrity of the finished surface is of great interest to qualify the quality of the workpiece in manufacturing processes. For that, surface roughness and surface burrs are important characteristics of the surface quality. In addition, the evaluation of the surface quality can optimize the manufacturing process parameters [10,11,12]. Moreover, it can be emphasized that in the cutting process of polymer materials, the surface quality, dimensional accuracy, and chip formation will be affected by the changes in cutting parameters [13,14,15].

Although the micromachining of polymers is a relatively small topic in academic research compared to metals, certain applications provide a reasonable amount of work. In the case of processing different polymers, the researchers studied some process identification criteria, such as surface roughness, cutting force, and material removal rate. Early work on polymer processing includes the cutting model developed by Kobayashi and Saito [16] and the orthogonal cutting work by Gindy and Vickerstaff [17]. Aramcharoen et al. [18] proved that the micro-milling process has the capability of microfluidic chips with microchannels and micropillar arrays made of biocompatible polymer materials, which have good mechanical processing surface quality and a high aspect ratio of up to three. Xiao and Zhang [15] studied the machinability of typical thermoplastic polymers and emphasized the effect of material viscosity on surface integrity, chip formation, and cutting force. They pointed out that the optimal processing conditions must be based on the properties of the polymer, such as glass transition temperature, fracture toughness, and molecular mobility. Crabtree, etc. [19] proposed that mechanical processing below the glass transition temperature will produce a glass surface generated by the glassy response, which is characterized by low elongations. Due to the viscoelasticity of polymers, the same change in material properties can be achieved by controlling the rate of deformation. If the deformation is applied at a lower rate, the high deformation rate will cause the glassy behavior to become brittle, and rubber behavior will appear. Some researchers have suggested that the machinability and surface finish of many polymers can be optimized in the temperature range around the glass transition temperature [20,21]. Ghosh et al. [21] believed that mechanical processing in the rubber or glass state would result in poor surface finish. In the rubber state, the machined surface is determined by tearing and corrugation, while in the glass range, cutting chips and fracture of the workpiece surface are observed. The formation of polymer shear bands was studied by Davies et al. and found it also has a significant impact on the mechanical properties and morphology of the machined surface apart from damaging the cutting tools [22]. The local damage on the machined surface due to adiabatic shear bands also has been studied, which affects the required transparency for optical applications [23,24]. Due to the large elastoplastic deformation and thermomechanical coupling phenomenon, cutting polymers with high dimensional accuracy and surface quality is challenging. Chiu et al. [25] studied the crack trajectory during the cutting of PMMA based on linear elastic fracture mechanics. According to their results, the polymer exhibits complex viscoplastic behavior rather than pure linear elastic behavior. For example, it is found that PMMA will undergo significant strain hardening at low strain rates, and strong strain-softening will occur at higher strain rates [26]. Recently, great efforts have been made to describe the mechanical response of glassy polymers under a wide range of strain rates [27,28,29,30]. Generally, the processing properties of polymers mainly depend on their mechanical, thermal, and rheological properties. High thermal expansion and elasticity, as well as low thermal conductivity and softening temperature, often hinder effective material removal in precision machining. Material characteristics are also very sensitive to temperature, and unnecessary material behavior irregularities in the cutting area will be generated during processing.

To summarize some of the studies reported in polymer processing, most studies contribute significantly to the relationship between surface quality and processing parameters without considering the properties of the polymer. Although the study of burr formation and minimization is well established in various forms of metal or brittle material machining, very little work has reported the occurrence of burrs during polymer micro-milling. The objective of this work is to bridge these gaps by establishing a link between machining parameter settings, burr formation, and cutting chip characteristics. Dynamic mechanical analysis (DMA) experiments and fast micromilling full factor experiments were performed on the samples. The machinability of three kinds of polymers, PMMA, PEEK, and PI, subjected to micromilling with different spindle speeds, feed rates, depth of cut, and tools is investigated. Through the dynamic mechanical analysis experiment, the glass transition temperature and temperature effect on the mechanical property of PMMA, PEEK, and PI are analyzed. Then, the micromilling experiment is conducted to show the characteristics of polymers during the high spindle speed micromilling process. This work can contribute to optimizing process parameters for polymer machining in microscale and ultra-precision machining.

## 2. Materials and Methods

### 2.1. Materials

**Figure 1:**
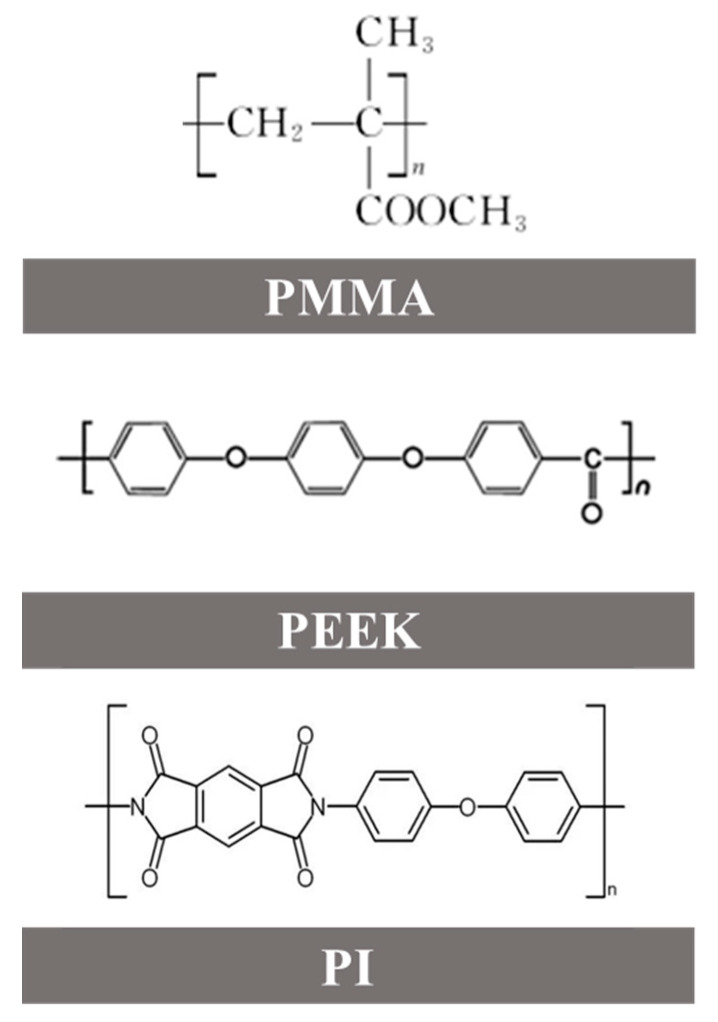
Chemical structures of poly(methyl methacrylate) (PMMA), polyetheretherketone (PEEK) and polyimide (PI).

Commercial grades of PMMA, PEEK, and PI used were casting bulk plates with a size of 30 mm × 20 mm × 8 mm, and the detailed parameters of the samples are shown in Table 1. The Chemical structures of PMMA, PEEK and PI are shown in Figure 1. The dynamic mechanical analysis experiment, the elastic modulus, and temperature effect on the mechanical properties of PMMA, PEEK, and PI during the micromilling process are studied in this work.

### 2.2. Design of Experiments

#### 2.2.1. Dynamic Mechanical Analysis (DMA)

In this work, the DMA experiment was carried out in a TA Instruments DMA Q800 dynamic mechanical analyzer at a frequency of 1 Hz in the cantilever mode. The experiments were performed on dry samples of prismatic shape with a size of 50 mm × 50 mm × 5 mm. The temperature dependence of storage modulus and loss tangent were studied from 30 °C up to 180 °C with a heating rate of 2 °C/min with a frequency of 1 Hz.

#### 2.2.2. Micro Milling Test

**Figure 2:**
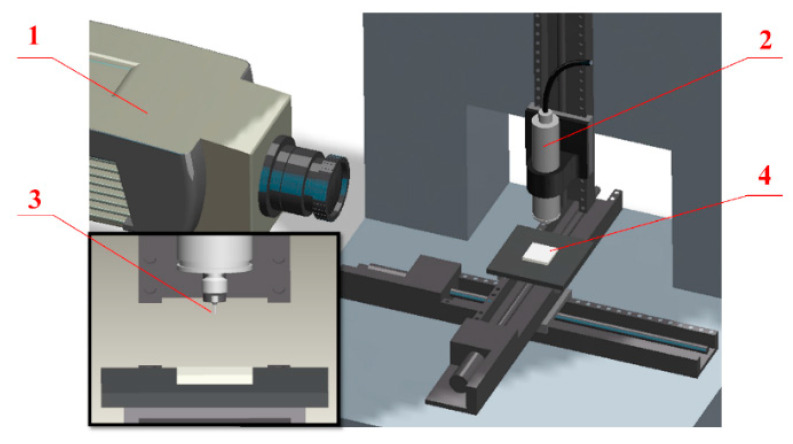
Experimental setup. (**1**) infrared camera, (**2**) spindle, (**3**) milling cutter, (**4**) workpiece.

A high-precision micromilling setup was used in this work (Figure 2), and it included a high-speed electric spindle (NAKANISHI, E3000), three linear precision translation stage (Puai Nano Displacement Technology, Co., Ltd., M304, Shanghai, China), and the infrared camera (IGA 6 Advanced, Lumasense IMPAC). The ultra-fine particle cemented carbide micromilling cutter (Mitsubishi Japan, purchased from Misumi China, model MS2SS) was applied as the machine tool with a diameter of 0.5 mm and 1 mm. The micromilling parameters for workpiece machineability analysis are displayed in Table 2. Moreover, the machining process was conducted without using any cutting fluid, and the room temperature was kept at 20 °C.

For the sake of eliminating the influence of tool superposition wear on the machined surface, each group of the experiments was conducted with a new micromilling cutter. The infrared camera was applied to measure the temperature of the milling zone. It can show a heat point near the cutting zone, and the temperature near the cutting zone can be calculated based on the material emissivity.

## 3. Results and Discussions

### 3.1. Dynamic Property Analysis

**Figure 3:**
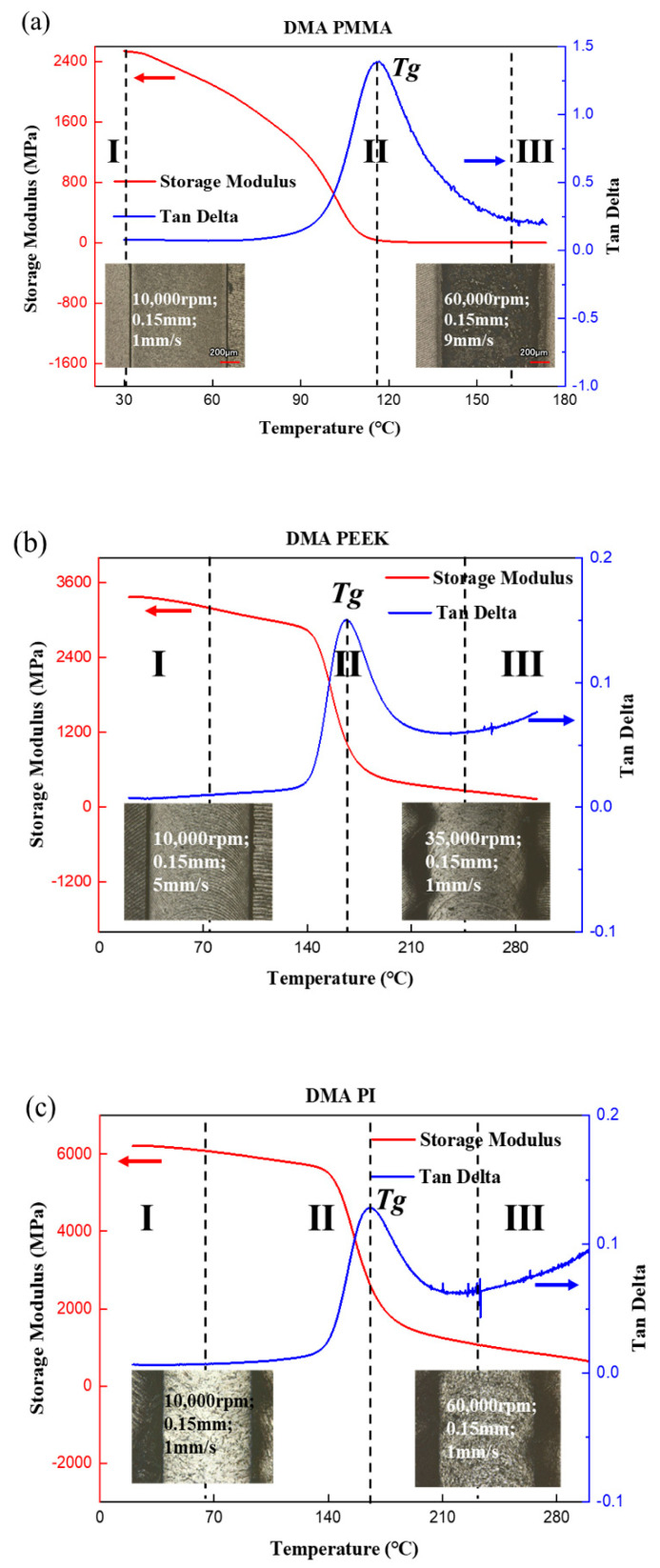
Dynamic properties (loss factor (tan(δ)) and storage modulus (E′), three distinct regimes: (I) glass state (II) viscoelastic (III) rubbery) vary with temperature, (**a**) dynamic mechanical analysis (DMA) of PMMA, (**b**) DMA of PEEK, (**c**) DMA of PI.

Dynamic mechanical analysis (DMA) was used to determine the mechanical properties of a viscoelastic material, which was also referred to in this paper as the viscoelastic parameters. The temperature-dependent loss factor (tan(δ)) and storage modulus (E′) curves of polymers are shown in Figure 3. The blue line was the relationship between tan(δ) and temperature. The red line represents how the storage modulus varied with the temperature. In general, with the increasing temperature, the storage modulus decreased until the temperature reached the glass transition temperature (*Tg*), and then the storage modulus kept constant. For the tan(δ) curve, it increased until the temperature reached *Tg* and then decreased. The glass transition temperature *Tg* of different could be found, and it was the peak of the tan(δ) curve. Three distinct regimes: (I) glass state (II) viscoelastic (III) rubbery could be defined based on the DMA results and surface quality of the grooves.

According to the results, the *Tg* for three kinds of PMMA, PEEK, and PI are 116 °C, 167 °C, and 166 °C, respectively. Three distinct regimes, such as the glassy regime, viscoelastic regime, and the rubbery regime, could be found, and different groove structures are shown in Figure 3a–c. As shown in Figure 3a, when the processing temperature was higher than 160 °C, which was larger than *Tg*, obvious burrs were observed along the groove. The DMA results of PEEK are shown in Figure 3b, and the *Tg* of PEEK was about 167 °C, which was much higher than that of PMMA. When the temperature was at about 70 °C, the edge of the groove was neat without obvious burrs. If the processing temperature were about 240 °C, which is near the *Tg* of PEEK, the groove shape of the PEEK sample would be ruined. The DMA characteristics of PI were similar to that of PEEK according to the results displayed in Figure 3c. The *Tg* of PI was about 166 °C, and the groove edge quality was also very good. With the increase of temperature, the edge shape was ruined, and big burrs or sintered edges could be observed. On the whole, when the polymers were in the glass state, the material could be removed in a brittle way. With the temperature rising, the polymers were in the viscoelastic or rubbery state, big burrs could be found on the groove edges, and the material was melted or burned from the bulk material.

### 3.2. Characteristics of Processing Temperature in Micromilling Process

**Figure 4:**
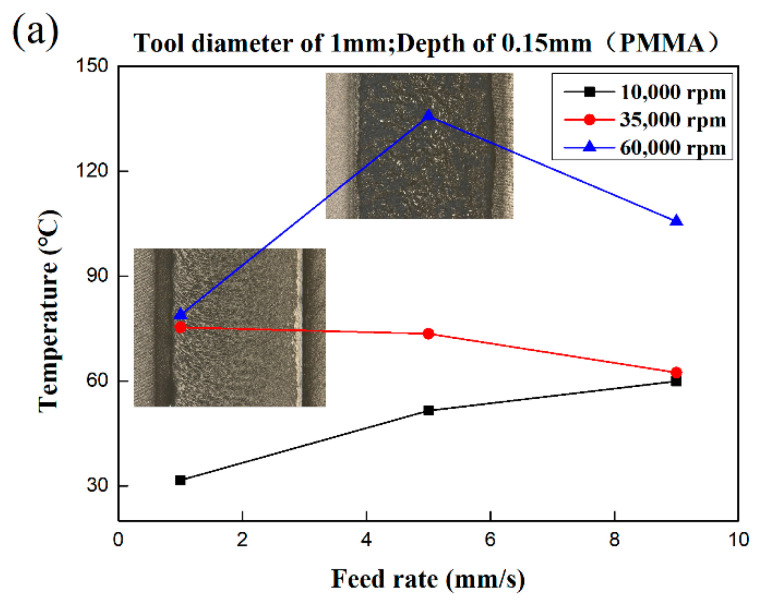

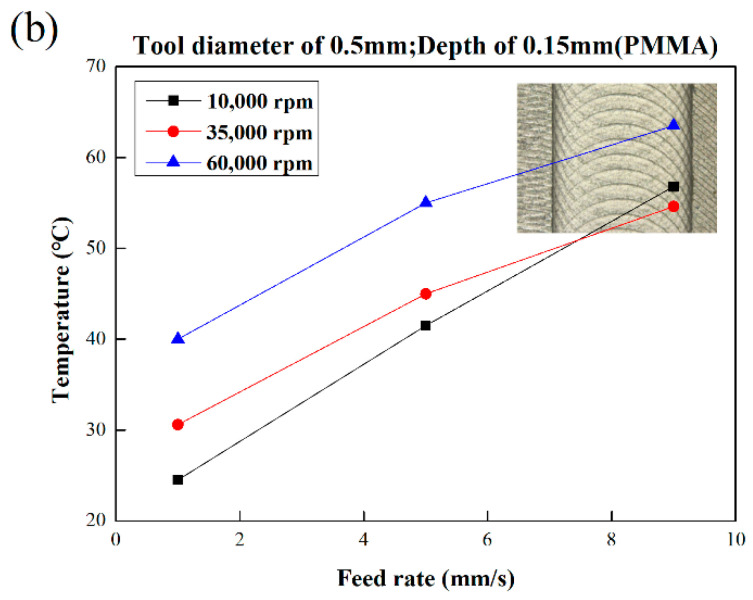
Bottom morphology of PMMA at various process parameters: (**a**) tool diameter 1 mm; (**b**) tool diameter 0.5 mm.

**Figure 5:**
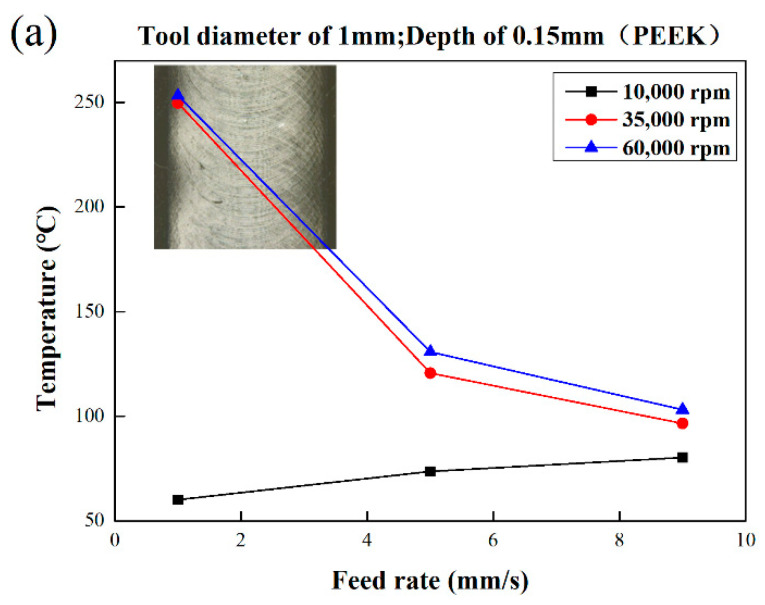

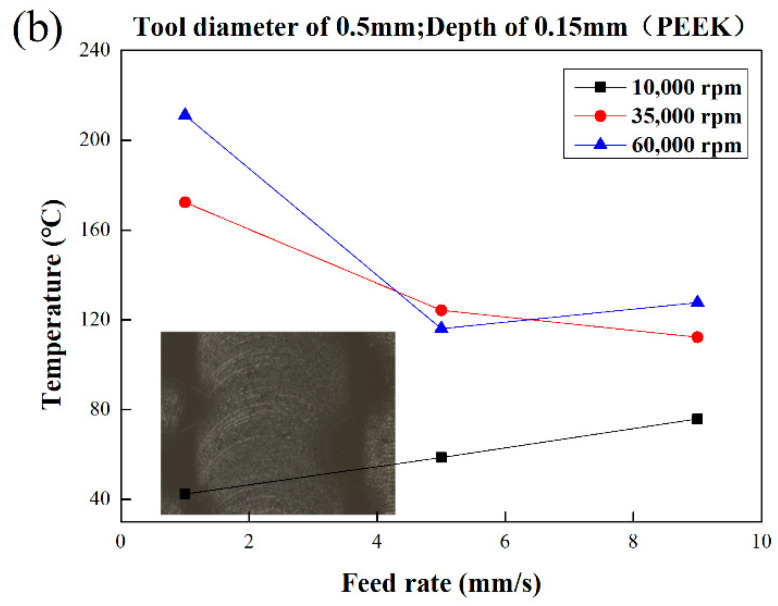
Bottom morphology of PEEK at various process parameters: (**a**) tool diameter 1 mm; (**b**) tool diameter 0.5 mm.

**Figure 6:**
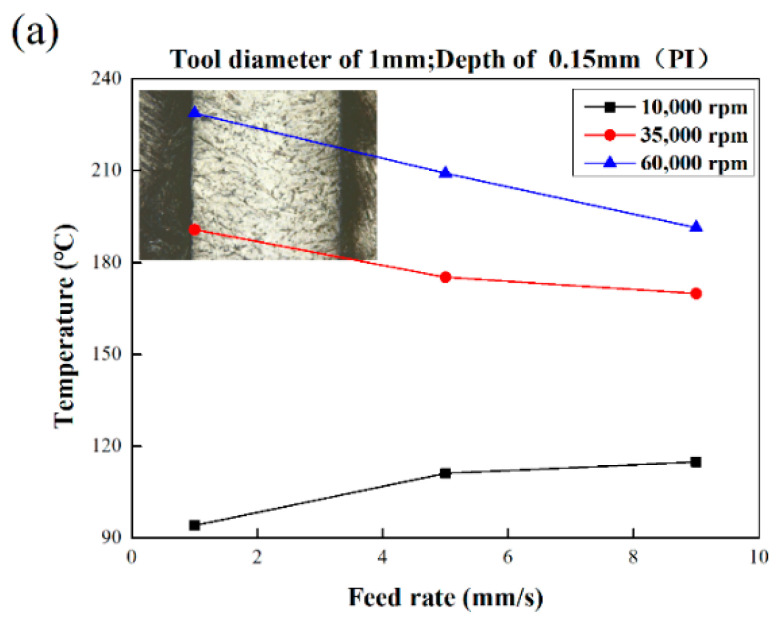

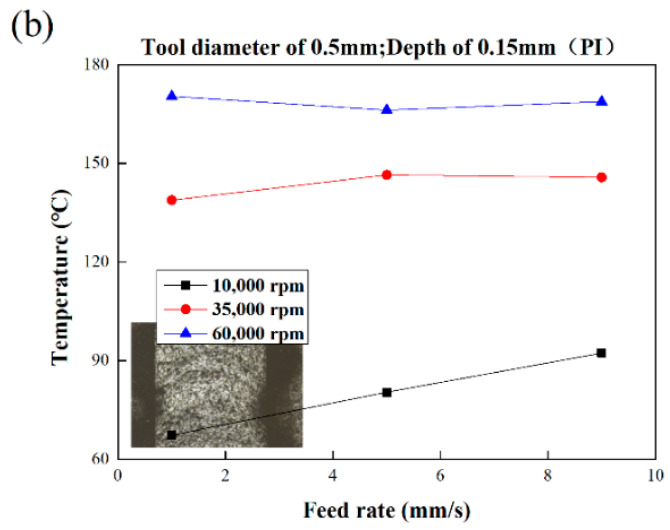
Bottom morphology of PI at various process parameters: (**a**) tool diameter 1 mm; (**b**) tool diameter 0.5 mm.

The relationships of milling processing temperature and processing parameters for three workpieces are shown in Figure 4, Figure 5 and Figure 6. The black, red, and blue lines represented three spindle speeds: 10,000 rpm, 35,000 rpm, and 60,000 rpm, respectively. It can be observed that the milling temperature of the three materials increased with the increase of the spindle speed. The groove bottom morphology was displayed under certain points in figures, and different material removal characteristics can be observed.

As shown in Figure 4, the PMMA was milled with a cutter (0.5 mm) under different parameters. The minimum temperature observed was only 24.5 °C, and the overall temperature was maintained below 70 °C with obvious tool marks at the bottom of the groove. Under this circumstance, the brittleness removal mechanism plays a leading role. When a milling cutter in the size of 1 mm was applied for the milling experiment, the minimum temperature was 31.6 °C, and the maximum temperature could reach 135.7 °C. Compared with the results of the milling cutter in 0.5 mm, the temperature had increased significantly. When the spindle speed was 10,000 rpm, the temperature still showed a tendency to increase with the rising of the feed speed, and it reached 59.9 °C when the feed speed was 9 mm/s. However, with the spindle speed of 35,000 rpm, the milling temperature decreased with increasing feed rate with a difference of about 12.9 °C. It should be noted that the feed speed was 1 mm/s at this rotation speed, and the bottom of the material was softened with a temperature of 75.3 °C. With the spindle speed of 60,000 rpm, the temperature increased first and then decreased with the increase of the feed rate, and the maximum temperature was 135.7 °C with the feed rate of 5 mm/s. The surface quality was very poor and showed obvious characteristics of high viscoelasticity. In general, the viscoelasticity of the material increased, and the material was changed to the viscoelastic state with the increasing temperature.

As shown in Figure 5, the milling temperature of PEEK material was higher than that of PMMA, especially since the temperature increased significantly at high-speed. Two sizes of milling cutters were applied in this part, and the temperature increased with the increase of feed rate at the spindle speed of 10,000 rpm. The temperature increased from 42.5 to 75.9 °C with the milling cutter in 0.5 mm, and the temperature of the milling cutter in 1 mm increased from 60.2 °C to 80.3 °C with a slight increase. When the spindle speed reached 35,000 rpm and 60,000 rpm, the temperature showed a clear trend of decreasing with the increase of feed rate. When milling with a 0.5 mm cutter, the maximum temperature was 211.2 °C with the spindle speed of 60,000 rpm and the feed speed of 1 mm/s. In the case of milling with a 1 mm cutter, the maximum temperature was about 253.2 °C under the same parameters. Compared with PMMA, the morphology of the bottom of the PEEK material did not change significantly with temperature. In Figure 5, there was no obvious change in the shape of the groove, but the burrs are too high, and most of the bottom surface is blocked by them. Since the temperature changed a lot, it can be roughly inferred that the material removal characteristics may be changed.

The processing temperature of the PI is much higher than that of PMMA, as displayed in Figure 6. Even at the lowest temperature observed in this group of experiments, there is no obvious stage of brittleness removal similar to PMMA. The milling temperature of PI material was also higher than that of PMMA and was closer to the milling temperature of PEEK. When using a 1 mm cutter for milling, the milling temperature tended to decrease as the feed rate increases (from 94.1 °C to 114.8 °C) with the speed of 10,000 rpm. When the speed increased to 35,000 rpm and 60,000 rpm, the milling temperature decreased with the rising of feed rate, and the drop rate was gentler than that of PEEK with a maximum temperature of 228.8 °C. When using a 0.5 mm milling cutter for milling, at a speed of 10,000 rpm and 35,000 rpm, the milling temperature increased slightly with the increase of feed rate. While at a speed of 60,000 rpm, the milling temperature was in the range from 165 to 170 °C. Compared with the milling results of PMMA and PEEK, the groove bottom of PI after milling had no obvious tool marks even at low temperatures. No obvious brittle removal characteristics could be found, but the viscoelastic characteristic is more obvious at a higher temperature.

On the whole, the milling temperature generally shows a gradually increasing trend with the increase of the feed rate at the lower temperature stage (lower than 120 °C). At this stage, the materials applied in this work exhibited brittle property. They have a large storage modulus, a small loss tangent value, and a small loss modulus ratio. If the feed rate was increased, the number of polymer chain breaks per unit time increased, and more energy released by material separation, and the temperature increased finally.

With the increase of the spindle speed and the tool diameter, the milling temperature further increased. When the temperature was near the *Tg*, the storage modulus of the material further decreased, and the loss tangent value increased at first and then decreased. The material was more like a flexible elastomer and was in the viscous flow state if the temperature was near the *Tg*. At this stage, the intermolecular fluidity of the polymer material increased, which resulted in the proportion of brittle removal during the milling process decreased. In addition, the material removal and molecular chain breakage are accompanied by plastic extrusion deformation and intramolecular losses. Therefore, with the increase of the feed rate, the temperature will tend to decrease. The polymer was a kind of time-dependent material. Under certain temperature and external force, it took a certain amount of time for the polymer molecule to change from one equilibrium state to another equilibrium state. During high-speed milling, the time was not long enough for the polymer to change the state before the material was removed. Therefore, the temperature will be different from the DMA test results to some extent.

### 3.3. Characteristics of Edge Burrs in the Micromilling Process

**Figure 7:**
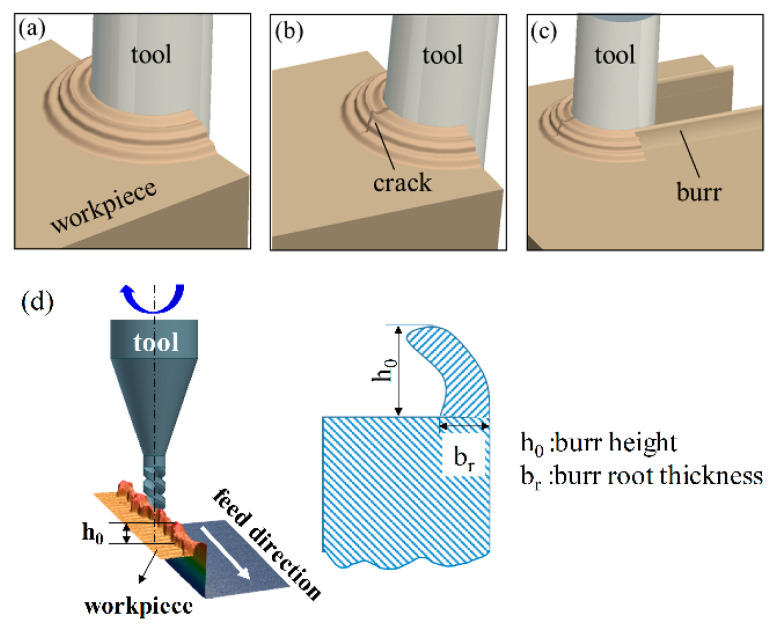
Modeling of burr formation during micro end milling: (**a**–**c**) The milling process of polymer, (**d**) the schematic of burr height and burr root thickness.

The burr formation was of particular importance in micromilling, which would limit the applications of polymers. For example, the burrs delimit the sealing capacity of joint microcomponents or seal ring performance. If the burrs were too high, a thick gap might result between the main parts, and liquids may leak. The burr was defined as an unwanted amount of material arising from plastic deformation on a workpiece edge. This extra material extended beyond the ideal workpiece edge and may be pendulous or associated with possible waveforms. The heights of burrs generated at the sideward were discussed, as is shown in Figure 7.

No chips were formed during the engagement of the micro end mill. The workpiece material was pressed on the tool to form a plastic deformation zone (Figure 7a). Continuing the process, a crack was formed near the center of the groove (Figure 7b). The forward movement of the cutting tool also caused burrs to form on the side edges. These burrs accumulated on the tool path until they are removed by the cutting tool or until further plastic deformation occurs or cracks form. The formation of burrs is described by the ratio of the feed per tooth to the thickness bf of the newly emerged burr root. If the feed amount of each tooth is constant and greater than the thickness of the produced burr root, no burrs will accumulate during this stage. A small number of burrs were reserved and were transferred to the flank.

**Figure 8:**
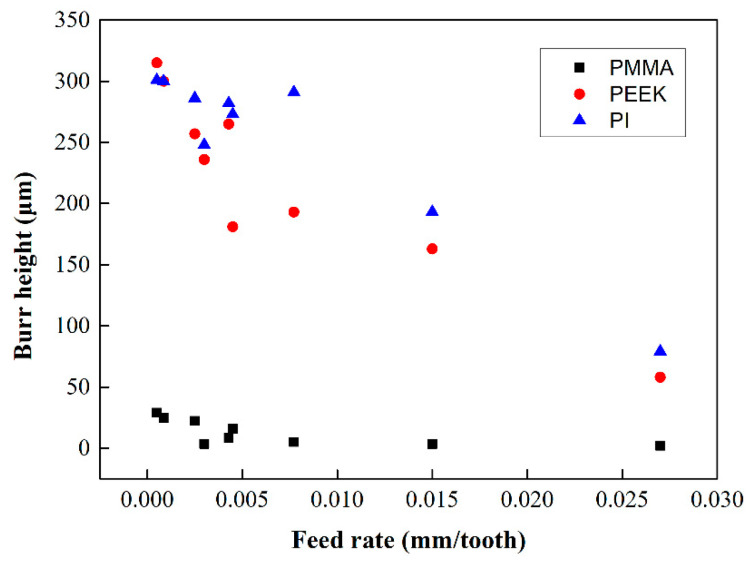
Burr height of the grooves for different polymers (tool diameter of 0.5 mm).

The characteristics of the burr height of various micro-milled polymers were discussed in Figure 8. The burr analyzed in this work was defined as Poisson burr, which was caused by the material squeezed at the cutting edge. In general, the burr height decreased with the increase in the feed rate. The milling burr of PMMA was in the range of 0–50 μm, which was significantly smaller than the burr produced by PEEK and PI (70–350 μm). Moreover, the burr heights of PEEK and PI were similar to each other. According to the results of PMMA, if the feeding rate was smaller than 0.01 mm/tooth, the burr height decreased sharply with the increase of feed rate. This was in line with the conclusions of Reichenbach’s work [3,6] that the burr accumulates when the feed per tooth is greater than the thickness of the burr root.

### 3.4. Characteristics of Cutting Chips in the Micromilling Process

**Figure 9:**
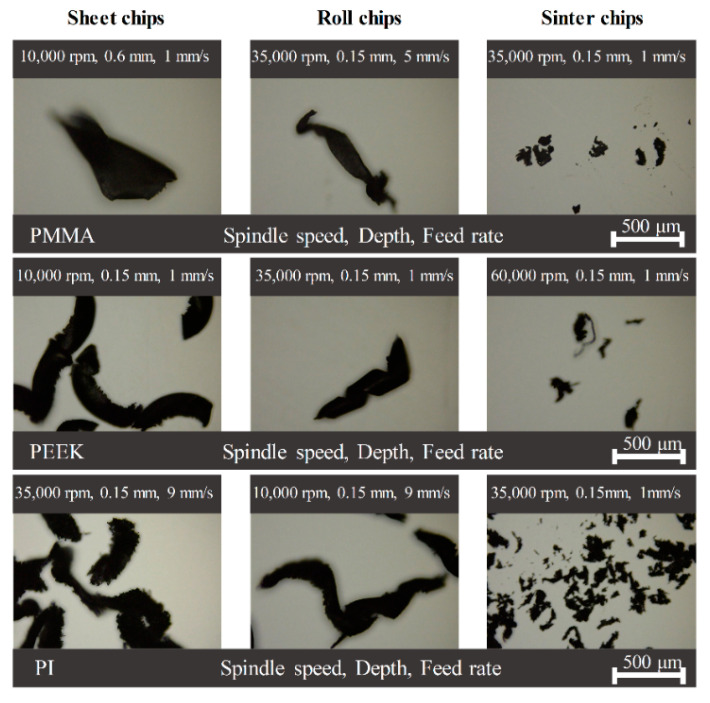
Characteristics of cutting chips for PMMA, PEEK, and PI.

Cutting chips generated during milling PMMA, PEEK, PI under different parameters were collected and observed under a laser scan confocal microscope (LSCM, VK-X250, Keyence Co., Osaka, Japan). As shown in Figure 9, the cutting chips were classified into three kinds according to the shape: sheet chips, roll chips, and sinter chips.

According to the results, the shape of the cutting chips was determined not only by spindle speed, cutting depth, or feed rate but also was determined by the comprehensive effect of processing parameters. For the PMMA sample, the cutting chips were in the sheet and sinter shapes with the same feed rate of 1 mm/s but different spindle speed (10,000 rpm, 35,000 rpm) and cutting depth (0.6 mm, 0.15 mm). However, for the PEEK sample, the cutting chips changed from the sheet shape to the roll shape and sinter shape with the increasing of spindle speed. As for the PI sample, the sinter cutting chips were generated with a smaller feed rate under the same spindle speed and cutting depth.

**Figure 10:**
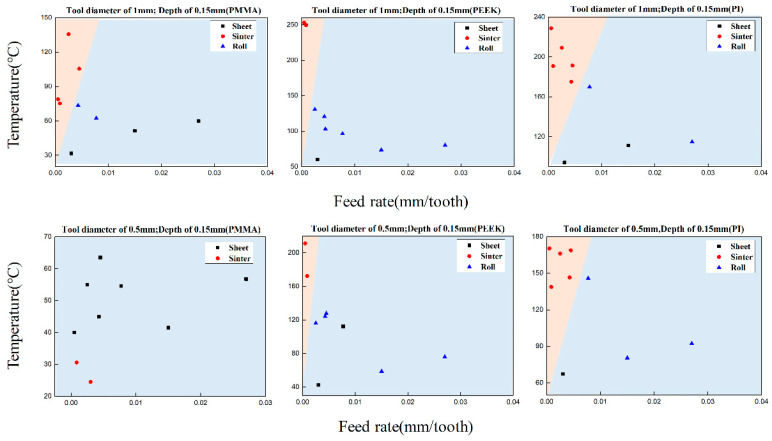
PMMA, PEEK, PI chip distribution with temperature and feed rate.

The cutting chips distribution of PMMA, PEEK, PI with temperature, and feed rate are shown in Figure 10, and two distinct regimes in pink and blue were classified according to the cutting chip shapes. The cutting chips in the pink regime were in the sinter shape, and the cutting chips were in the sheet and roll shape in the blue regime. The red circles represent the sinter chips, the blue triangles represent the roll chips, and the black squares represent the sheet chips. In general, the sintered chips are concentrated in regimes with lower feed rates and higher temperatures. When the feed rate is high and the temperature is low, the chip forms appear as sheet chips and roll chips.

When using a milling cutter with a diameter of 1 mm to mill three materials, the temperature of PMMA sintered chips was the lowest of the three. When the temperature was only 75.3 °C, the sinter chips were generated with a feed rate of 0.857 μm/tooth. The PEEK was milled with the same parameters, and the lowest temperature of such sintered chips was 249.7 °C with a feed rate of 27 μm/tooth. However, under other milling parameters, most of them were roll chips, and just a small part of the chips was in the sheet shape with a temperature of 60.2 °C. PI began to generate sintered chips at the temperature of 175.2 °C with a feed rate of 4.286 μm/tooth.

For the situation milling with the 0.5 mm cutter, the milling temperature of PMMA material was low, and no sintered chips were generated. Most of the chips were in the sheet shape, and just a small part of the chips was in the roll shape. The lowest temperature of the sintered chips in PEEK material was 172.4 °C, which was higher than the other two materials with the feed rate of 27 μm/tooth, and most of the other parameters were still in a roll shape. The sintered chips of PI were generated at a temperature of 138.8 °C with a feed rate of 15 μm/tooth.

Above all, the PI sample was easier to generate sintered chips under the same process parameters with the two sizes of milling cutters. The processing temperature of PEEK was higher than the other two materials, but the temperature declined quickly with the increase of feed rate since the thermal conductivity was higher than the other two materials. The sinter chips of PI just could be observed under the condition of small feed rate and high-temperature. PMMA showed good machinability when milled with a 0.5 mm cutter, and the temperature was kept between 24.5 °C and 63.5 °C. It is also found that the material removal characteristics change with the milling processing parameters and temperature. The increase in temperature results in the material from a brittle state to a viscoelastic state or a viscous fluid state at higher temperatures. Moreover, the chip morphology changes from sheet to roll or sintered chips.

## 4. Conclusions

In this paper, milling experiments and DMA tests were carried out on three kinds of polymer materials, PMMA, PEEK, and PI, under various processing parameters. The milling characteristics of each material were comprehensively analyzed, including the surface quality, burr generation, cutting chip characteristics, and material removal characteristics at different temperatures.

(1) The removal characteristics of polymer materials will change with processing temperature and milling parameters. In this work, the highest milling temperature of the three materials was about 240 °C, and the lowest one was about 25 °C. The material will be removed by brittle fracture at low temperatures. However, the material will be in the viscoelastic state with the increasing temperature and be removed in a ductile way. The surface quality of the brittle removal is generally better than that of the viscoelasticity state;
(2) The average size of PMMA burrs is much smaller burr than that of PEEK and PI under the same conditions. For example, when milling with a 0.5 mm cutter, the burr height of PMMA is within 50 μm. However, the burr height of PEEK and PI was up to two or three hundred micrometers. According to the results, the decrease in the feed rate will promote the generation of burrs. This is because when the feed rate is less than the thickness of the burr root, residual material will accumulate together, which is the top burr;
(3) Three kinds of cutting chips could be observed under different cutting parameters. When the feed rate is small, and the temperature is high, the material is in viscoelasticity. The material is more likely to burn by the friction of the cutting tool, and sintered chips are generated with bad surface quality. At the lower temperature, the material is removed in the brittle mode with sheet and roll shape cutting chips. This work is of great value in analyzing the removal mechanism of polymer materials and optimizing process parameters.

## Figures and Tables

**Table 1:** Mechanical properties of polymers used in this work.

Sample	PMMA	PEEK	PI
Manufacture	DX001, Mitsubishi Chemical Polymer Nantong, China	SP-21, Shenzhen Dongqilai Plastic Material Co. LTD.	PEEK-1000, Dongguan Yusen Industrial Co., LTD.
Density (g/cm^3^)	1.14	1.51	9
Modulus of elasticity (GPa)	3.2	2.895	4.3

**Table 2:** Micromilling parameters for workpiece machineability analysis.

Factor	Group 1	Group 2	Group 3
Spindle speed (rpm)	10,000	35,000	60,000
Feed rate (mm/s)	1	5	9
Tool diameter (mm)	0.5/1	0.5/1	0.5/1

## Data Availability

The data presented in this study is openly available.
